# Mechanism Influencing the Drying Behavior of Bitumen Emulsion

**DOI:** 10.3390/ma14143878

**Published:** 2021-07-12

**Authors:** Chun Li, Jian Ouyang, Fangjie Dou, Jingtao Shi

**Affiliations:** 1 PetroChina Fuel Oil Limited Company Research Institute, Beijing 100195, China; li-chun@petrochina.com.cn (C.L.); sjt0822@163.com (J.S.); 2 School of Transportation and Logistics, Dalian University of Technology, Dalian 116024, China; 3 Foshan Gaofu Petrochina Fule & Asphalt Co., Ltd., Foshan 528531, China; doufangjie@petrochina.com.cn

**Keywords:** bitumen emulsion, drying behavior, emulsion quality, maximum packing fraction of bitumen droplets, bitumen droplets size

## Abstract

The drying process of bitumen emulsion largely dominates the strength development of emulsion-based mixtures for pavement structure, thus it can be used to judge the quality of bitumen emulsion. However, the drying behaviour of bitumen emulsions was seldom considered. The emulsion drying and film formation theory are employed to study the drying process of different bitumen emulsions with a thin layer. Results indicated the drying process of bitumen emulsion can be divided into three stages: (a) an initial high evaporation rate stage; (b) an intermediate stage with a rapidly decreasing evaporation rate; (c) a final stage with a very small evaporation rate. The boundaries among the three stages can be identified by studying the water evaporation rate. Three drying parameters, i.e., the critical volume fractions of bitumen defining the boundaries among the three stages and the maximum packing fraction of bitumen droplets, are proposed to quantitatively characterize the drying behavior of bitumen emulsion. High values of these parameters indicate a bitumen emulsion that has rapid drying behavior. Therefore, these parameters are independent of the emulsifier type, but they are highly dependent on the bitumen’s droplet size. These drying parameters increase with a decrease in bitumen droplet size. Therefore, bitumen emulsion with a smaller size distribution of bitumen droplets can have a more rapid drying behavior, which is recommended in real engineering.

## 1. Introduction

As traditional pavement materials, bitumen emulsion-based materials, which can be paved at ambient temperature, are widely used in pavement maintenance, preservation, and rehabilitation. With the growing environmental awareness worldwide in recent years, bitumen emulsion-based materials are gradually more preferred due to their merits of low energy consumption and carbon emission compared to traditional hot bitumen mixture [1,2]. Although the benefits of bitumen emulsion-based materials are evident, their application has great constraints because of long-time curing. As a typical feature, the precondition for recovering the adhesive ability of bitumen emulsion is that most of the water should be evaporated or consumed. In this regard, it is of great importance to deeply understand the drying behavior of bitumen emulsion as well as its corresponding mixtures.

Because of the constraint of long-time curing, to promote the technologies of bitumen emulsion-based materials, many scholars focused on how to accelerate the strength development of bitumen emulsion mixture [3,4,5,6,7]. Generally, the water consumption rate of the bitumen emulsion mixture is related to the curing condition. Low humidity and high temperature conditions can greatly increase the water evaporation rate, thus shortening the curing time to reach the desired strength [3,4,5,6]. However, this curing condition cannot be realized in real engineering. Therefore, cement hydration can also consume some water in the mixture, thus adding cement, even rapid-hardening cement, can be greatly beneficial to the early strength of the bitumen emulsion mixture [3,4]. However, the rapid-hardening cement can be definitely harmful to the chemical stability of bitumen emulsion so that the workable time of the bitumen emulsion mixture may be greatly shortened [8]. Emulsifier content can affect the chemical stability of bitumen emulsion, thus it can also affect the strength development of bitumen emulsion mixture. At lower emulsifier contents, the premature breaking of emulsion easily occurs so that the bitumen emulsion mixture can have a certain strength soon, however, it’s mechanical performance in the later curing stage may be inferior due to poor adhesion of bitumen at the interface to the aggregate [7]. Therefore, bitumen type, bitumen and water content can affect the strength of the mixture as well as its development process [2,7,9]. For instance, a bitumen emulsion mixture with the harder bitumen can have a higher indirect tensile strength [9]. However, the bitumen type is chosen according to the climate and traffic grade in engineering, and the optimum bitumen and water content should be used to ensure the best mechanical performance of the bitumen emulsion mixture [2]. Therefore, bitumen type, bitumen and water content are seldom adjusted in the application after knowing the optimum bitumen and water content. Overall, no efficient methods can be currently used in engineering to accelerate the strength development of bitumen emulsion mixture.

Essentially, the adhesive ability of bitumen emulsion is highly related to its residual water content. Thus, the drying behavior of bitumen emulsion as well as its corresponding mixtures, which can greatly dominate the strength development of bitumen emulsion mixture, is being gained concerns recently. In the aspect of the drying of bitumen emulsion mixture, Saadoon et al. investigated the water evaporation process of bitumen emulsion mixtures with different types of cement [10,11]. They found that the Marshall stability of the bitumen emulsion mixture had a good relationship with its water evaporation. Therefore, although the addition of cement (especially for rapid hardening cement) can greatly increase the early Marshall stability of the bitumen emulsion mixture, it cannot shorten the drying process of the mixture [11]. Thus they believed that bitumen emulsion was the main component influencing the drying process of the mixture [10]. In the aspect of pure emulsion drying, Goavec et al. investigated the water concentration distribution in bitumen emulsion during drying through Magnetic Resonance Imaging [12]. Results indicated that non-uniform distribution of bitumen droplets in the vertical direction could be observed during drying. The concentration of bitumen droplets near the surface can be higher than that near the bottom. When the concentration of bitumen droplets can reach the maximum packing fraction of bitumen droplets, skin phenomenon can occur during drying [13]. Ouyang et al. discussed the reasons for the non-uniform distribution of bitumen droplets during drying and proposed a drying condition to ensure the uniform distribution of bitumen droplets during drying [14]. Therefore, they also recommended that the quality of bitumen emulsion can be evaluated by its drying behavior [14].

The above studies are very meaningful to understand the drying behavior of pure bitumen emulsion and bitumen emulsion mixture as well as their effects on the strength development of bitumen emulsion mixture. However, these studies are all based on a bitumen emulsion. No studies focused on the drying behavior of different emulsions. Therefore, the mechanism influencing the drying behavior of different bitumen emulsions is unclear. As a result, it is unclear how to produce fast-drying bitumen emulsion. Therefore, the drying of bitumen emulsion is seldom considered in real engineering. According to the current specifications [15,16], the types of bitumen emulsion are mainly classified by the breaking grade, such as rapid, medium and slow setting. Meanwhile, the quality of bitumen emulsion is mainly evaluated by the bitumen content in emulsion and the basic technical properties of the residual bitumen in the engineering. Because of no requirements for the drying process of bitumen emulsion, mixtures with different bitumen emulsions qualified by current requirements may have significantly different strength development processes, especially for the early strength.

Based on the above consideration, the objectives of this research are as follows:

- To quantitatively study the drying behavior of different bitumen emulsions;
- To evaluate the quality of bitumen emulsions from the drying aspect;
- To reveal the key properties of bitumen emulsion dominating its drying behavior.

## 2. Materials and Specimens Preparation

### 2.1. Materials

**Figure 1:**
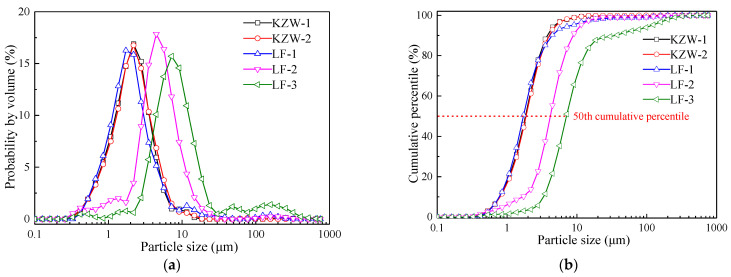
The particle size distribution of bitumen emulsions, (**a**) Differ ential distribution, (**b**) Cumulative percentile.

Self-made cationic bitumen emulsions were tested to achieve the objectives of this study. Five slow-setting bitumen emulsions were fabricated in the laboratory by the same basic formula in which emulsifier, water and bitumen contents are 4%, 36% and 60%, respectively. Paving grade base bitumen 60/80 is widely used in the production of bitumen emulsion in pavement engineering in China, which was also used in this study, with its general properties as well as its chemical components listed in Table 1. The first two emulsions were produced by an emulsifier (coded as KZW) from Tianjin Kangzewei Co., Ltd. in Tianjin, China. The other three emulsions were produced by an emulsifier (coded as LF) from Shanghai Focusen Pavement Engineering Co., Ltd. in Shanghai, China. By adding small doses of different stabilizers, the five emulsions can have different particle size distributions of bitumen droplets, which can be shown in Figure 1. The bitumen particle size distribution was measured by a laser particle size analyzer (Mastersize 2000, Malvern Instruments Ltd., Malvern, UK). Based on Figure 1b, the bitumen droplet size *r*_50_ (droplet diameter at the 50th cumulative volume percentile) can be obtained in Table 2. Therefore, the main difference of different emulsions with the same emulsifier is the droplets size distribution. The five emulsions can be used to study the effect of the emulsifier and bitumen droplet size distribution on the drying behavior of bitumen emulsion.

### 2.2. Drying Test

**Figure 2:**
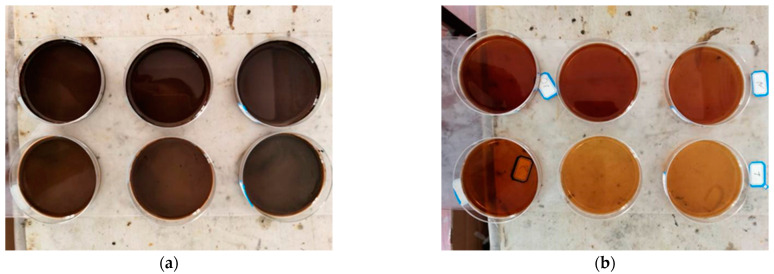
Drying test of asphalt emulsion and emulsifier solution, (**a**) Asphalt emulsion, (**b**) Emulsifier solution.

The drying process of the bitumen emulsion was investigated by the gravimetric measurement of the mass loss due to water evaporation. As shown in Figure 2, cylindrical plastic containers of 52 mm in diameter and 15 mm in height were used in the drying test. According to the drying theory of latex, the concentration distribution of droplets may be non-uniform in the latex, especially for the thicker layer. In that case, skin formation easily occurs during drying [17]. According to previous studies [14,18], an ideal, typical drying curve of non-skin emulsion, drying can be obtained for the emulsion specimens with the thickness of 3 mm and the initial bitumen content of 40%, thus these specimen conditions were also employed in this study. Bitumen emulsions were diluted to emulsions with bitumen content at 40% by deionized water before the test. To guarantee the accurate layer thickness of specimens, the masses of specimens were calculated according to the theoretical volume and density of bitumen emulsion [14]. The drying test was performed in a temperature-controlled environmental chamber at 25 °C. Meanwhile, the airflow speed and relative humidity around the place of test specimens were around 0.4 m/s and 38%. The water loss of specimens was measured in regular time intervals ranging from 0.4 h to 1 h by a balance with the precision of 10^−4^ g. The drying test for every specimen was performed with three repetitions.

The mass per unit area (expressed in g/m^2^) was used to express the emulsion drying process. The bitumen volume fraction (*ϕ*) gradually changes during drying, which can be calculated by Equation (1):

(1) $$\phi =\frac{V_{B}}{V_{B}+V_{W}}=\frac{m_{B,\mathrm{unit}}/\rho_{B}}{m_{B,\mathrm{unit}}/\rho_{B}+\left(m_{W,\mathrm{unit}}-m_{\mathrm{loss}}\right)/\rho_{W}}$$

where *V*_B_ and *V*_W_ are the volumes of bitumen and residual water, respectively; *m*_B,unit_ and *m*_W,unit_ are the mass of the bitumen and initial water by unit area, respectively; *m*_loss_ is the loss of the water by unit area (g/m^2^); *ρ*_B_ and *ρ*_W_ are the density of bitumen and water, respectively; *ϕ* is the bitumen droplets volume fraction. Therefore, the average evaporation rate between two adjacent test points can be calculated by Equation (2).

(2) $${\dot{E}}_{i\sim i+1}=\frac{m_{\mathrm{loss},i+1}-m_{\mathrm{loss},i}}{t(i+1)-t(i)}$$

where *Ė*_i~i+1_ is the average evaporation rate between test points of *i* and *i*+1; *m*_loss,i_ and *m*_loss,i+1_ are the corresponding loss of the water by unit area, respectively; *t*(*i*+1) and *t*(*i*) are the corresponding time of the test points of *i* and *i*+1, respectively.

### 2.3. Observation Test of Bitumen Droplets Packing during Drying

**Figure 3:**
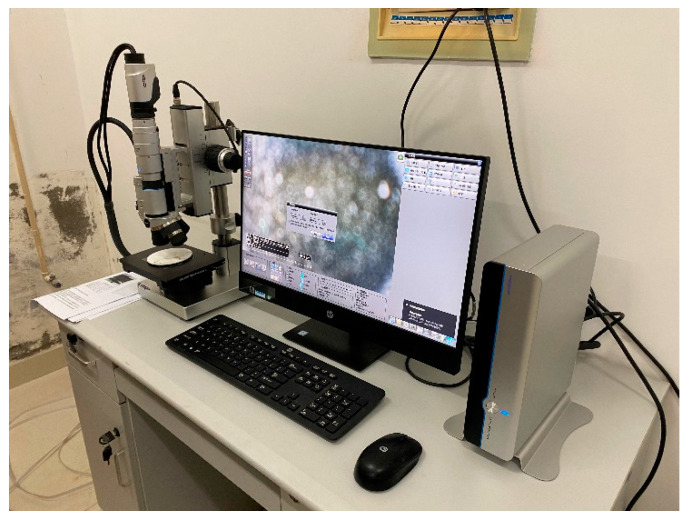
Three-dimensional digital microscope.

A three-dimensional digital microscope with maximum magnification at 5000× shown in Figure 3 was employed to record the bitumen droplets packing state during drying. As an optical microscope, the three-dimensional digital microscope can directly obtain the digital image of specimens without any treatment. The employed magnification was 700× in this study. A very thin film of diluted bitumen emulsion was tested to clearly observe the bitumen droplets packing state in this test. 

## 3. Methodology to Evaluate the Emulsion Drying

### 3.1. Theory and Qualitative Description of the Emulsion Drying

**Figure 4:**
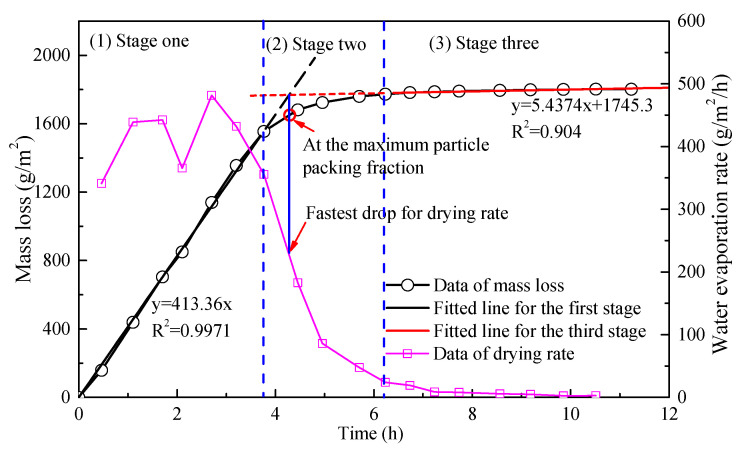
Drying process of bitumen emulsion (LF-1).

**Figure 5:**
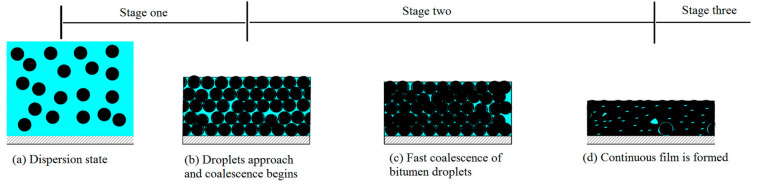
Schematic representation of the three-stage drying process.

A typical drying process of bitumen emulsion is shown in Figure 4. It can be seen from Figure 4 that the drying process of bitumen emulsion can be roughly divided into three stages. According to the basic theory of the drying and film formation process of emulsion [17,18,19,20], the drying behavior of bitumen emulsion is highly related to the bitumen droplets packing state, which can be described as followed:

1. The first stage corresponds to the droplets in the semi-diluted regime when the concentration of the droplets is far from the maximum particle packing fraction in Figure 5a. The bitumen emulsion has a free evaporation surface thus its evaporation rate can be equal to that of pure water or dilute emulsifier solution. This regime ranges from the initial state, *ϕ*_0_, to the critical volume fraction of droplets, *ϕ*_1_, at which bitumen droplets approach and the irreversible coalescence of bitumen droplets may begin as Figure 5b.
2. The second stage corresponds to the compact regime when the increase in the volume fraction of bitumen by evaporation beyond *ϕ*_1_ takes place with the further packing, deformation and irreversible coalescence of bitumen droplets. The evaporation rate sharply decreases in this stage because the area of the evaporation surface is getting smaller. This process is determined by the flow out of the water from the gap among the compact droplets until they coalesce to a roughly continuous film as Figure 5d. In this stage, there is a moment such as that demonstrated in Figure 5c when bitumen droplets reach the maximum packing state.
3. The final stage corresponds to the diffused regime when the bitumen molecules are inter-diffused among droplets, thus becoming the continuous bitumen phase with the inclusions of the aqueous phase in Figure 5d. The residual water in the film escapes either by diffusion through the capillary channels between the coalescing droplets or extrusion due to the reconfiguration of the bitumen phase. In this stage, the evaporation rate is much smaller and tends to zero.

### 3.2. Quantitative Analysis of the Drying Process

According to the typical drying process of bitumen emulsion mentioned previously, it can be easily inferred that the key parameters dominating the drying process are the average evaporation rate of the first stage (*Ė*_0_), the critical droplet volume fraction between the first and second stage (*ϕ*_1_), the maximum particle packing state (*ϕ*_m_), and the critical droplet volume fraction between the second and third stage (*ϕ*_2_). Bitumen emulsion can have a high evaporation rate from the initial droplet volume fraction (*ϕ*_0_) to *ϕ*_1_ and *ϕ*_m_, thus higher values of *ϕ*_1_ and *ϕ*_m_ indicate that bitumen emulsion can be dried to a denser state at a high evaporation rate equal to *Ė*_0_. Accordingly, bitumen emulsion has a very low evaporation rate in the third stage, thus the third drying stage can be shortened if *ϕ*_2_ is high.

To obtain the key drying parameters for quantitatively analyzing the drying process of bitumen emulsion, the boundaries among different stages should firstly be identified. According to previous studies [14,18], the boundaries could be identified by studying the water evaporation rate. As shown in Figure 4, the water evaporation rate firstly undulates and then decreases sharply with the drying time, and finally slowly tends to zero. The first boundary, in which the later evaporation rate decreases sharply compared to the average evaporation rate before this point, can be observed. Similarly, the second boundary, in which the later evaporation rate decreases very slowly, can be found. After identifying the two boundaries, the corresponding water loss can be obtained. Accordingly, the values of *ϕ*_1_ and *ϕ*_2_ can be calculated by Equation (1). Meanwhile, the initial evaporation rate, which is the average evaporation rate of the first stage, can be also calculated.

Determination of *ϕ*_m_ is more difficult than *ϕ*_1_ and *ϕ*_2_. Theoretically, there is no obvious droplets deformation and coalescence about bitumen droplets before the drying time of *ϕ*_m_, but a significant droplets deformation and coalescence are occurred after this moment [17,20]. Therefore, the area of evaporation surface and the connection channels of water in emulsion are significantly changed in the drying time of *ϕ*_m_. It can be inferred that the change in the evaporation rate at the point of *ϕ*_m_ should be maximum in the drying curve. Since the data of water loss is recorded in regular time intervals ranging from 0.4 h to 1 h, the accurate curve of the drying rate is unknown. Therefore, as shown in Figure 4, the cross point of the fitted lines of the first and third stages is used to estimate the drying time of *ϕ*_m_ according to our previous studies [14,18]. Then, the corresponding water loss can be accurately estimated from the curve of mass loss versus drying time. After knowing the water loss, the maximum packing fraction of bitumen droplets can be calculated according to Equation (1).

Based on the above quantitative calculation process, the key drying parameters of the specimen in Figure 4 can be calculated in Table 3.

## 4. Results about the Drying Process of Different Emulsions

**Figure 6:**
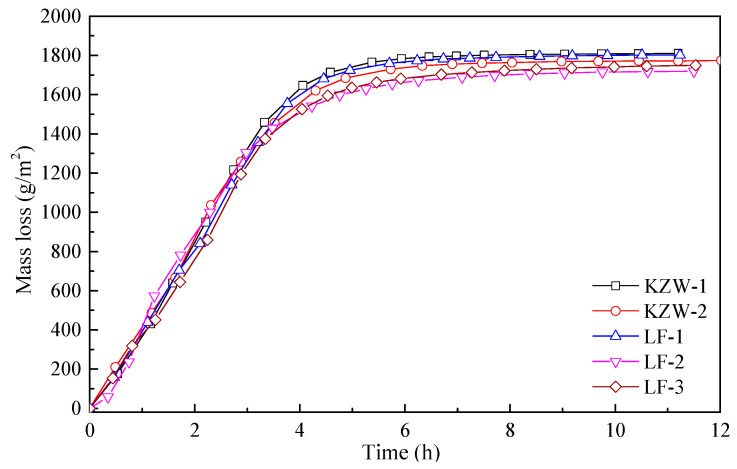
Drying process of different emulsions.

**Figure 7:**
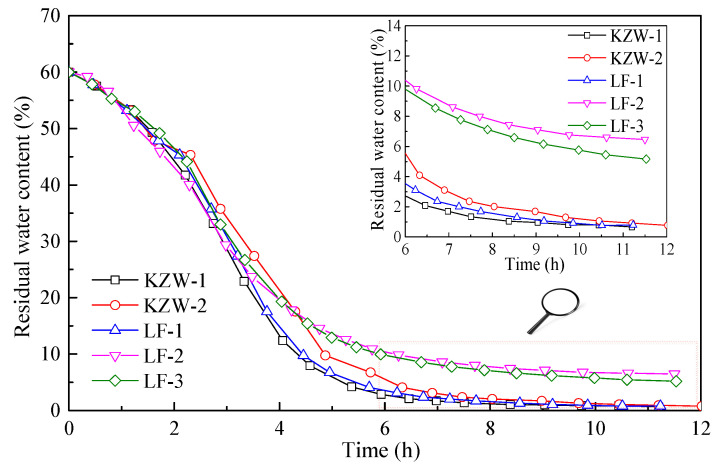
Residual water content of different emulsions during drying.

The drying processes of different bitumen emulsions are shown in Figure 6. It can be seen from Figure 6 that all five bitumen emulsions have a typical three-stage drying process. The mass loss of the five emulsions differs little at the first stage, however, it differs greatly at the third stage. To better clearly show the drying behavior of different emulsions, the residual water content of emulsions during drying is calculated in Figure 7. It can be seen from Figure 6 that the residual water content of different emulsions differs greatly at the third stage. The residual water content of emulsions is ranked as: LF-2 > LF-3 > KZW-2 > LF-1 ≈ KZW-1. Since the water evaporation rate is very slow in the third stage, the difference in the residual water content indicates that the time for recovering the adhesive ability differs greatly for the five emulsions. For instance, LF-2 and LF-3 have very large values of residual water content. As a result, it can be a long process for recovering their adhesive ability. On the contrary, KZW-1 and LF-1 can quickly recover their adhesive ability. Therefore, even for different emulsions with the same emulsifier, the drying process can be also different. It should be stated here that the five emulsions can have the same breaking grade and residual properties if they are evaluated by the current specifications. However, it can be inferred according to Figure 7 that they may have significantly different drying and adhesion forming properties if they are applied in engineering. Therefore, it is necessary to evaluate the quality of bitumen emulsion by its drying behavior.

Based on the above quantitative analysis method of the emulsion drying process, the drying parameters of different emulsions can be calculated in Table 4. It can be seen from Table 4 that the initial evaporation rate of five emulsions ranges from 403 g/m^2^/h to 438 g/m^2^/h. The small relative difference (the maximum value is 8.7%) nearly has no effect on the drying behavior of the first stage. However, the three key drying parameters (*ϕ*_1_, *ϕ*_m_ and *ϕ*_2_) differ greatly for different emulsions. For instance, the value of *ϕ*_2_ can range from 91.22% to 97.91% for the five emulsions. As a result, the residual water content ranges from 2.09% to 8.78% for the five emulsions at the beginning of the third drying stage. Since the evaporation rate is very low in the third drying stage. A little difference in *ϕ*_2_ can significantly affect the drying process of emulsions. LF-1 and KZW-1 have high values of *ϕ*_1_, *ϕ*_m_ and *ϕ*_2_, thus their drying process is relatively short compared to other emulsions. LF-2 and LF-3 have low values of *ϕ*_1_, *ϕ*_m_ and *ϕ*_2_, thus a very long time is required to reach their desirable adhesive ability. Therefore, the drying parameters of *ϕ*_1_, *ϕ*_m_ and *ϕ*_2_ can effectively and quantitatively reflect the drying process of bitumen emulsion. A good quality bitumen emulsion should be dried quickly to recover its adhesive ability after placement. A previous study indicated that the *ϕ*_m_ for a given bitumen emulsion was a constant parameter that differed little with the initial bitumen concentration and drying conditions [12]. Therefore, the drying parameters of *ϕ*_1_, *ϕ*_m_ and *ϕ*_2_ can be reasonable indexes for evaluating the quality of bitumen emulsion.

## 5. Discussion

As mentioned above, the drying behavior of bitumen emulsion can reflect its quality. Quick-drying bitumen emulsion is more preferred in pavement engineering. In this regard, factors dominating the drying behavior of bitumen emulsion will be studied based on the five emulsions in the following section.

### 5.1. Effect of Emulsifier on the Drying Behaviour of Bitumen Emulsion

**Figure 8:**
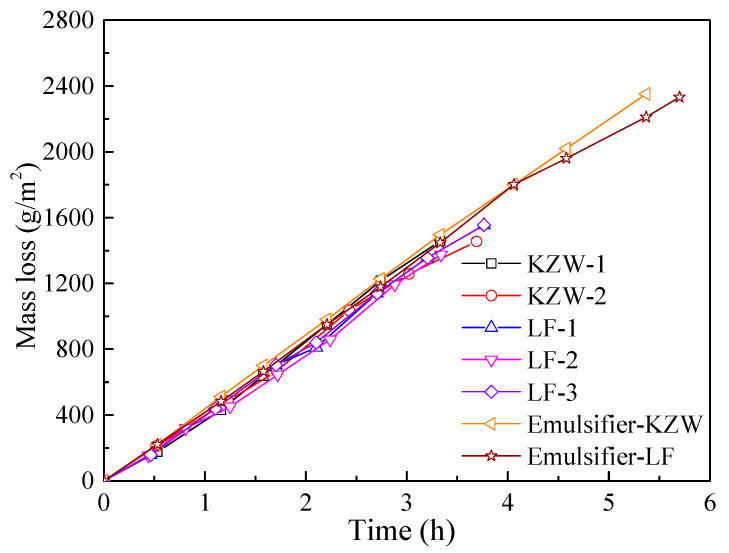
The first drying stage of emulsions and their emulsifier solutions.

**Figure 9:**
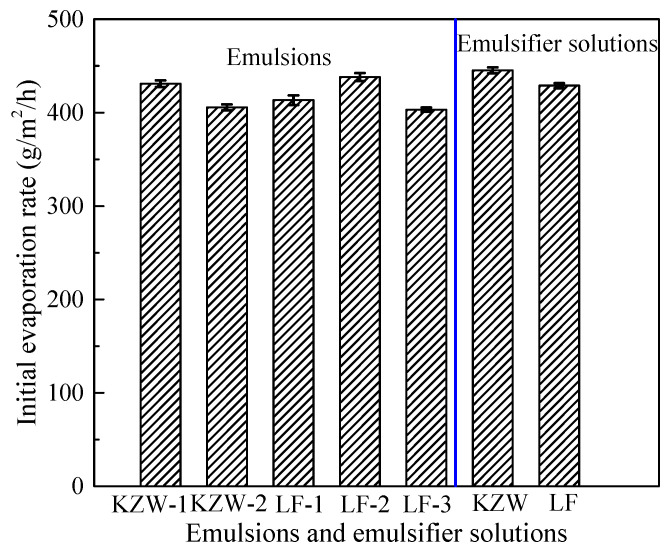
Initial evaporation rate of emulsions and their emulsifier solutions.

The first drying stage of bitumen emulsions and their emulsifier solutions are shown in Figure 8. The emulsifier content in the emulsifier solution is 4.4%, which is almost equal to the emulsifier concentration in the diluted bitumen emulsion. Based on Figure 8, the initial evaporation rates of bitumen emulsions and their emulsifier solutions are calculated in Figure 9. It can be seen from Figure 9 that the evaporation rate of different emulsifier solutions and emulsions differs slightly. Their relative difference can be lower than 5%. Therefore, the emulsifier types do not affect the evaporation rate of bitumen emulsion. Actually, the evaporation rate of emulsifier solution is nearly equal to that of water, which mainly depends on the drying condition, i.e., temperature, humidity and air speed.

Emulsifier not only cannot affect the initial evaporation rate of bitumen emulsion, but also cannot affect its drying parameters of *ϕ*_1_, *ϕ*_m_ and *ϕ*_2_. It can be seen from Table 4 that KZW-1 and LF-1 can have high values of *ϕ*_1_, *ϕ*_m_ and *ϕ*_2_, but parameters of *ϕ*_1_, *ϕ*_m_ and *ϕ*_2_ are relatively low for KZW-2, LF-2 and LF-3. Therefore, emulsions produced by both emulsifiers may be of good or bad quality in drying. Overall, the drying behavior of bitumen emulsion is independent on the emulsifier type.

### 5.2. Effect of Droplet Size on the Drying Behavior of Bitumen Emulsion

**Figure 10:**
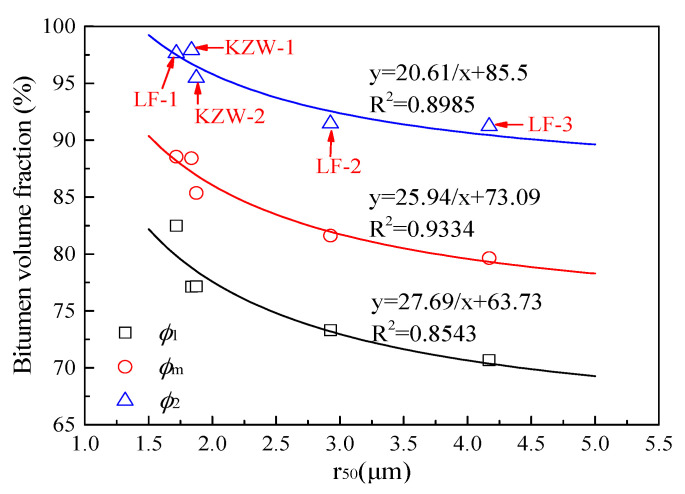
Relation of drying parameters (*ϕ*_1_, *ϕ*_m_ and *ϕ*_2_) with *r*_50_ for the five emulsions.

As mentioned previously, the initial evaporation rate differs little for different bitumen emulsions, but the three drying parameters (*ϕ*_1_, *ϕ*_m_ and *ϕ*_2_) differ greatly. Therefore, the drying parameters (*ϕ*_1_, *ϕ*_m_ and *ϕ*_2_) dominate the drying process of bitumen emulsion. The relations of the drying parameters (*ϕ*_1_, *ϕ*_m_ and *ϕ*_2_) with bitumen droplets size are shown in Figure 10. Since bitumen droplets in emulsion have a wide size distribution. Droplet size *r*_50_ (droplet diameter for the 50th cumulative volume percentile) is employed as a representative particle size in Figure 10. It can be seen from Figure 10 that all of the three drying parameters are decreased with the increasing bitumen droplets size. Bitumen emulsion with a smaller droplet size can be dried more quickly. Therefore, decreasing the bitumen droplets’ size can improve the drying quality of bitumen emulsion.

Essentially, the three parameters (*ϕ*_1_, *ϕ*_m_ and *ϕ*_2_) are all indexes about bitumen droplets packing state during drying. Thus, it is reasonable that the three parameters are all related to bitumen droplets’ size. Equation (3) is used to fit the relation of the three parameters with *r*_50_. It can be seen from Figure 10 that the three parameters (*ϕ*_1_, *ϕ*_m_ and *ϕ*_2_) have a positive correlation with 1/*r*_50_. Especially for *ϕ*_m_, the correlation coefficient can be higher than 0.9. Therefore, the bitumen droplet size can greatly dominate the drying behavior of bitumen emulsion.

(3) $$\phi_{m}=\frac{a}{r_{50}}+b$$

where *a* and *b* are fitting parameters.

### 5.3. Mechanism Analysis about the Drying Parameters

**Figure 11:**
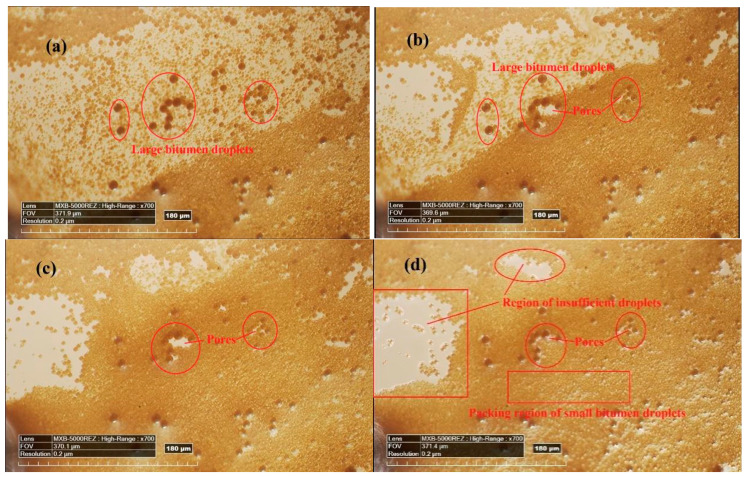
Evolution process of bitumen droplets packing during drying. (**a**) Most of droplets are well dispersed and water is enough; (**b**) Droplets packing begin in water- deficient region; (**c**) Large droplets can affect the small droplets packing during droplets packing; (**d**) Final droplets packing state with little free water.

As mentioned above, the drying parameters (*ϕ*_1_, *ϕ*_m_ and *ϕ*_2_), which dominate the drying process of bitumen emulsion, are highly related to the bitumen droplets’ size. To analyze the possible mechanism for this phenomenon, the evolution process of the bitumen droplet’s packing during drying is studied by a three-dimensional digital microscope, which is shown in Figure 11. It can be seen from the four images that the motion of small bitumen droplets is very active so that they can be densely packed. However, large bitumen droplets almost stay in the same position during the whole droplets packing process due to their weak motion ability. As a result, large bitumen droplets are acted as obstacles during the packing process of small bitumen droplets. Because of this obstacle effect, as shown in Figure 11c,d, pores are normally formed around large bitumen particles at the end of the particles packing process. Therefore, large bitumen droplets are harmful to the bitumen droplets packing state. It is reasonable that the three drying parameters (*ϕ*_1_, *ϕ*_m_ and *ϕ*_2_) decrease with the increasing bitumen droplet’s size in Figure 10.

Overall, the packing state of the bitumen droplets is related to the motion ability of bitumen droplets in the emulsion. The motion ability of bitumen droplets in the emulsion is highly related to the bitumen droplet size. Smaller bitumen droplets can have higher motion ability in the emulsion. Essentially, the motion ability of particles in a dilute suspension is due to the particle Brownian motion. According to the Stokes–Einstein relation [18], the particle Brownian motion can be evaluated by the particle diffusion coefficient (*D*) in Equation (4).

(4) $$D=\frac{kT}{3\pi \eta r}$$

where *kT* is the thermal energy; *r* is the particle diameter; *η* is the medium viscosity. It can be seen from Equation (4) that the particle diffusion coefficient has a positive correlation with 1/*r*. Since the three drying parameters (*ϕ*_1_, *ϕ*_m_ and *ϕ*_2_) depend on the motion ability of the bitumen droplets (i.e., the diffusion coefficient of the bitumen droplets), it is reasonable that these parameters have high correlations with 1/*r*_50_ in Figure 10.

## 6. Conclusions

In this paper, the drying behavior of different bitumen emulsions is studied. Quantitative drying parameters are proposed to characterize the drying process of bitumen emulsion according to the drying theory of emulsion, and factors influencing the drying process of bitumen emulsion are discussed. Based on the results and discussion, the following conclusions can be drawn:

1. The drying process of bitumen emulsion, as well as the corresponding bitumen droplets packing evaluation process, can be divided into three stages: (a) an initial high evaporation rate stage in which bitumen droplets are well dispersed; (b) an intermediate stage in which bitumen droplets are contacted, deformed and further coalesced so that the evaporation rate rapidly decreases; (c) a final stage with a continuous bitumen film in which the evaporation rate is near-constant but much smaller than that of the first stage. The boundaries among the three stages can be identified by studying the water evaporation rate.
2. The initial evaporation rate differs little for different emulsions, thus the key parameters affecting the drying process of bitumen emulsion are the critical volume fractions of bitumen defining the boundaries among the three stages and the maximum packing fraction of bitumen droplets (i.e., *ϕ*_1_, *ϕ*_m_ and *ϕ*_2_). Bitumen emulsion with higher values of *ϕ*_1_ and *ϕ*_m_ can be dried to a denser state at a high evaporation rate. Bitumen emulsion with a higher value of *ϕ*_2_ has low residual water content when the third drying stage begins. Therefore, high values of the drying parameters (*ϕ*_1_, *ϕ*_m_ and *ϕ*_2_) indicate that bitumen emulsion has a good drying behavior.
3. The emulsifier type has little effect on the initial evaporation rate and the key drying parameters (*ϕ*_1_, *ϕ*_m_ and *ϕ*_2_) of bitumen emulsion. The key drying parameters (*ϕ*_1_, *ϕ*_m_ and *ϕ*_2_) of bitumen emulsion are highly related to the bitumen droplet size. They are all decreased with the increasing bitumen droplet size. The mechanism for this phenomenon is that the three drying parameters (*ϕ*_1_, *ϕ*_m_ and *ϕ*_2_) are essentially indexes about the bitumen droplets packing state during drying. Due to Brownian motion, small bitumen droplets can have high motion ability, thus they can easily be densely packed. Overall, decreasing the bitumen droplet size can improve the drying quality of bitumen emulsion.

## 7. Future Work

The key drying parameters are proposed to evaluate the quality of bitumen emulsion, and the mechanism influencing the key drying parameters is analyzed. However, the direct index of the quality of bitumen emulsion is its film formation properties (e.g., the adhesive ability and its recovery rate, dynamic modulus and its recovery rate). Therefore, the film formation properties and their relationship with the key drying parameters should be investigated for better evaluating the quality of bitumen emulsion.

## Figures and Tables

**Table 1:** Properties of the base bitumen.

Property	Test Specifications	Value
General properties		
Penetration at 25 °C (0.1 mm)	ASTM D 5	72
Softening point R and B (°C)	ASTM D 36	47.0
Ductility at 15 °C (cm)	ASTM D 113	>100
Kinetic viscosity at 60 °C (Pa s)	ASTM D 2171	227
Flash point (°C)	ASTM D 92	276
SARA composition	ASTM D 4124	
Saturates (mass fraction, %)		13.9
Aromatics (mass fraction, %)		41.5
Resins (mass fraction, %)		32.4
Asphaltenes (mass fraction, %)		12.4

**Table 2:** Bitumen droplet size at the 50th cumulative volume percentile.

Emulsion Code	KZW-1	KZW-2	LF-1	LF-2	LF-3
*r*_50_(μm)	1.837	1.874	1.718	2.926	4.170

**Table 3:** Key drying parameters of bitumen emulsion (LF-1).

Parameters	*Ė*_0_ (g/m^2^/h)	Time at *ϕ*_1_ (h)	Time at *ϕ*_m_ (h)	Time at *ϕ*_2_ (h)	*ϕ*_1_ (%)	*ϕ*_m_ (%)	*ϕ*_2_ (%)
Value	413.36	3.76	4.29	6.23	82.48	88.55	97.64

**Table 4:** Drying parameters of different bitumen emulsions.

Sample	*Ė*_0_(g/m^2^/h)	Time at *ϕ*_1_ (h)	Time at *ϕ*_m_ (h)	Time at *ϕ*_2_ (h)	*ϕ*_1_(%)	*ϕ*_m_(%)	*ϕ*_2_(%)
KZW-1	431.00	3.32	4.14	6.45	77.13	88.42	97.91
KZW-2	405.59	3.75	4.37	6.53	77.16	85.36	95.47
LF-1	413.36	3.76	4.29	6.23	82.48	88.55	97.64
LF-2	438.11	2.98	3.80	7.09	70.67	79.64	91.22
LF-3	403.2	3.34	4.05	6.69	73.31	81.61	91.46

## Data Availability

Data is contained within the article.
